# Evaluation of Ingestive Behavior, Ruminal and Blood Parameters, Performance, and Thermography as a Phenotypic Divergence Markers of Residual Feed Intake in Rearing Dairy Heifers

**DOI:** 10.3390/ani12030331

**Published:** 2022-01-29

**Authors:** Mayara Campos Lombardi, Hilton do Carmo Diniz Neto, Sandra Gesteira Coelho, Fernanda Samarini Machado, Luiz Gustavo Ribeiro Pereira, Thierry Ribeiro Tomich, Mariana Magalhães Campos

**Affiliations:** 1 Department of Animal Science, Veterinary School, Universidade Federal de Minas Gerais, Belo Horizonte 30161-970, MG, Brazil; mayaralombardi.vet@gmail.com (M.C.L.); hiltondinizneto@gmail.com (H.d.C.D.N.); sandragesteiracoelho@gmail.com (S.G.C.); 2 Brazilian Agricultural Research Corporation, Empresa Brasileira de Pesquisa Agropecuária-Embrapa Gado de Leite, Juiz de Fora 36038-330, MG, Brazil; fernanda.machado@embrapa.br (F.S.M.); luiz.gustavo@embrapa.br (L.G.R.P.); thierry.tomich@embrapa.br (T.R.T.)

**Keywords:** animal production, biological basis, feed efficiency, sustainability, water intake

## Abstract

**Simple Summary:**

The selection of highly efficient animals will support meeting the world’s future demand for products and food of animal origin. Thus, the identification of efficient animals and an understanding of the mechanisms inherent to this efficiency is fundamental for the progress of breeding systems. In the present study, we identify highly efficient animals for residual feed intake in dairy heifers. This animal category is unexplored in relation to this index. We utilized the classical parameters evaluated in cattle of different ages to carry out the study on these animals.

**Abstract:**

The objectives of this study were: (1) to identify and rank phenotypically divergent animals for residual feed intake (RFI) regarding their efficiency (high: HE or low: LE); (2) to evaluate their relationships with ingestive behavior, ruminal and blood parameters, performance, and infrared thermography; and (3) to determine if such measurements can be used as feed efficiency markers in rearing dairy heifers. Thirty-eight heifers, 143 d ± 4 (Mean ± SD) of age and 108.7 kg ± 17.9 of body weight were used. The animals were fed with a total mixed ration during the 91 d of the trial. A phenotypic divergence of DMI for RFI was observed between −0.358 and 0.337 kg/d for HE and LE, respectively. Dry matter intake (DMI) was lower in the HE (2.5 kg DMI/d vs. 3.1 kg DMI/d), as was the number of visits to the feed bin with consumption (59 vs. 71). Feed intake was the best predictor of said divergence. Water intake and number of visits to the feed bin were presented moderate correlations with RFI. The ruminal fermentation variables, blood metabolites, blood hormones (such as the other ingestive behavior variables), and infrared thermography were not able to accurately predict HE or LE animals.

## 1. Introduction

In the search for sustainability, animals that are more efficient at converting food into product are of unquestionable relevance [1,2]. The evaluation of feed efficiency can be obtained from measuring indexes such as feed conversion, average daily weight gain (ADG), residual body weight gain, residual feed intake (RFI), residual intake and body weight gain, or a combination of some of these indexes [3,4]. In this context, RFI appeared as a way to identify and classify animals in terms of their efficiency regarding consumption.

Due to the fact that this index is not based on weight at adult age, it allows for the selection of more efficient animals, not necessarily with greater weight but with lower maintenance energy. In addition, the fact that animals evaluated for RFI demonstrate a maintenance of their superiority at different stages of life indicates the future sustainable and economic potential associated with the selection by this index [5].

The RFI corresponds to individual feed intake, obtained from the difference between the observed DMI and the estimated DMI for each animal [3]. For the calculation of RFI, an approximate trial period of 90 d [6] was necessary. In beef cattle, RFI was associated with other variables such as blood metabolites and hormone concentrations, ruminal fermentation (pH, volatile fatty acids production), and superficial body temperature by using infrared thermography, microbial profile, and ruminal enzymes [7,8,9,10]. Most recently, lactating cows and preweaning calves have been the subject of RFI studies [5,11]. Nevertheless, postweaning calves in dairy cattle were not studied. This animal category was very important in the systems, and it needs to be evaluated. In the future, these results could be associated with the RFI in adult cows.

Literature shows DMI to be a well-established parameter to define divergence in RFI since the classification is dependent on consumption [3]. However, daily and individual measurements are impractical and costly. On the other hand, parameters such as ingestive behavior, ruminal fermentation and blood metabolites, or hormones presented controversial results [8,9,10,11]. Thus, the search for effective and more accessible parameters as predictors of efficiency in RFI continues [6]. As this is an unexplored category in RFI, the present study evaluates dairy heifers using the classic parameters found in the literature for the RFI study of cattle at different stages of life.

Understanding the behavior and role of these variables and their association with the phenotypic classification for RFI in dairy heifer can help develop more practical ways of identifying them daily. The aim of this study was to identify and rank dairy cattle heifers for RFI, to evaluate their relationships with ingestive behavior, ruminal and blood parameters, performance, and infrared thermography in Gyr heifers and to determine if such measurements can be used as feed efficiency markers during heifer rearing.

## 2. Materials and Methods

The experiment was conducted with heifers born and bred at the EMBRAPA Dairy Cattle Experimental Farm, Coronel Pacheco, Minas Gerais, Brazil. The experiment was conducted during the spring; temperature 33.7 °C ± 4.7 and humidity: 73 ± 9.56. After the trial period, the animals remained in their herd according to EMBRAPA regulations and will be utilized in further research.

### 2.1. Animals, Housing, and Management

A total of 38 Gyr heifers (143 d ± 4 d of age and 108.7 ± 17.9 kg of BW) was used. Immediately after birth, they were separated from their dams, moved to individual sand-bedded tie-stalls, weighed, and had their umbilical cords immersed in 10% iodine solution. Colostrum (10% of BW at birth; >50 g of IgG/L) was provided within 6 h after birth. During preweaning, calves received water and a starter (Soymax Rumen Pré-Inicial Floc-Total Alimentos, Três Corações, Minas Gerais, Brazil) at ease, and milk (42% of metabolic birth body weight). From 3 d of age, 8% chopped hay (*Tyfton* 85) was included. Weaning was at 87 d of age, and the animals stayed in their housing up to 101 d of age.

At 101 d of age, the heifers were transferred to a grass-covered paddock (*Cynodon dactylon*; 1350 m^2^) with a concrete area near the troughs, where they stayed during 28 d of adaptation and socialization. During the first seven days, their diet was 75% of the preweaning starter plus 25% of total mixed ration (TMR; 7% corn silage and 25% concentrate) (Table 1). In the second week, they were fed 50% of the preweaning starter plus 50% of TMR. In the third week, their diet consisted of 25% of the preweaning starter plus 75% of TMR, and in the last week 100% of TMR was fed to the heifers. Feed was supplied at will, ensuring a minimum 10% of leftovers.

Samples (0.5 kg) of corn silage and concentrate were collected three times a week then homogenized and packaged (at −20 °C). Feed samples were dried in a forced-ventilation oven at 55 °C for 72 h and then ground to 1 mm particle size in a Wiley Mill (model 3, Arthur H. Thomas Co., Philadelphia, PA, USA). Dry matter, crude protein, ethereal extract, and ashes were determined according to AOAC [12], and neutral detergent fiber was determined by the method of Van Soest [13]. Total digestible nutrients and metabolizable energy were calculated according to NRC [14]. Gross energy was determined using an adiabatic bomb calorimeter (IKA-C5000, IKA1 Works, Staufen, Germany).

### 2.2. Feed Intake, Water Intake, Body Weighing, Average Daily Gain, and Ingestive Behavior

The heifers were submitted to a trial period of 91 d in which they were fed with TMR twice a day at 08:30 and 15:30 h, and water was supplied at ease. The paddock was equipped with nine electronic feed bins (AF1000 JUNIOR Intergado Ltd., Contagem, Minas Gerais, Brazil) with a capacity of 55 kg and accuracy of ±0.025 kg and three electronic water bins (WD1000 Intergado Ltd., Contagem, Minas Gerais, Brazil) with a capacity of 40 kg of water and accuracy of ±0.025 kg. A body-weighing station (WD 1000 Intergado Ltd., Contagem, Minas Gerais, Brazil) with a capacity of 400 kg and accuracy of ±0.100 kg was coupled with each water bin so that to have access, the animals were kept at the weighing station. This technology has been tested and validated [15]. For the purposes of identification, each animal received an earring with an electronic transponder with an individual coding (FDX-ISSO 11784/11785; Allflex, Joinville, Brazil) before the trial period. 

Water and feed consumption were measured daily and individually as the difference in weight between the bin content at the beginning and end of the visit. Collection data were processed and stored by software from Intergado Ltd. Out of the total data collected in the 91 d of trial (intake, water intake and weighing), 16.5% were excluded due to occurrences unrelated to the equipment, such as days for maintenance of the paddock, heavy rain, and poor positioning of the animals on the body-weighing platform. Then 76 d were considered for consumption analysis and 90 d for weighing analysis, with 420 ± 87 weighing data being used for each animal. ADG was automatically calculated by software from Intergado Ltd. After the removal of the outliers, BW data were submitted to linear regression models, in which ADG was obtained from the first derivation of the selected model and calculated from the differences between the daily body weighing results. 

To evaluate ingestive behavior, the following data provided by software from Intergado Ltd. were used: total time at the bin (TTB), consumption time at the bin (CTB), number of visits to the feed bin with consumption (NVB), total time at the water bin (TTW), and number of visits at the water bin (NVW). Values of TTB lesser than 60 min/d and greater than 600 min/d were excluded from the analyses after pre-evaluation of the data since they were not within the normal values considering the average of time of all animals during the evaluation period. Values of TTW greater than 99 min/d were excluded for the same reason.

### 2.3. Residual Feed Intake

The RFI is the difference between the standardized daily DMI and the expected DMI. Therefore, the expected DMI was calculated using that is predicted based on animal growth and metabolic body as observed in the preestablished linear regression model [9]:

Yj = β0 + β1 (BW**0.75j) + β2 (ADGj) + ej

where:

Yj: standardized DMI of heifer j;
β0: is the regression intercept;
β1: is the regression coefficient on BW**0.75 to animal j,
β2: is the regression coefficient on ADG to animal j,
ej: is the associated error to animal j.

The classification of HE or LE was made according to the standard deviation (SD) around the mean value found for all animals (38): HE-RFI: <0.5 SD under the mean; *n* = 13, and LW-RFI: >0.5 SD above the mean; *n* = 14.

### 2.4. Rumen Variables and Analyses

A total of 100 mL of ruminal liquid was collected from each animal at 30, 60, and 90 d of trial, 3 h after the first feed, with the support of a flexible orogastric tube. The samples were sifted and filtered in double gauze. The pH was measured immediately with the aid of a portable potentiometer (DM-2-Digimed, São Paulo, Brazil). Samples were divided into two recipients: the first with 10 mL of ruminal liquid plus 2 mL of metaphosphoric acid (concentration 20%) for volatile fatty acid (VFA; acetate, propionate, and butyrate) concentration analysis; and the second with 10 mL of ruminal liquid plus 1 mL of sulfuric acid (50%) for analysis of ruminal ammoniacal nitrogen concentration.

Samples were centrifuged at 1800× *g* for 10 min at room temperature (22–25 °C) for measurement of VFA concentration using high performance liquid chromatography (Waters Alliance e2695 Chromatograph, Waters Technologies Brazil LTDA, Barueri, Brazil) with a C18 ODS 80A reverse-phase column separation system (150 × 4.6 mm × 5 µm). The conditions of analysis were an isocratic mobile phase consisting of 100% aqueous phosphoric acid solution, pH 2.35–2.55, oven temperature of 40 ± 5 °C, sample injection volume of 10 µL, run time of 20 min, and a detector with a wavelength of 210 nm. The ruminal ammoniacal nitrogen concentration was quantified after the distillation of Kjeldahl according to the INCT-CA N007/1 method [16] with boric acid solution (40 g/L), potassium hydroxide solution (132 g/L), and hydrochloric acid (0.49 g/L). After quantification, the adjustment required to use this method was performed according to INCT-CA N007/1 method.

### 2.5. Blood Metabolites and Hormones

Blood samples (30 mL) were collected from all the animals to determine metabolites and hormones at 30, 60, and 90 d, 3 h after the first feed of the day. The sample collections were carried out with local antisepsis (70% alcohol) by venous puncture of the jugular. Four siliconized vacuum tubes (Becton, Dickinson and Company, Franklin Lakes, NJ, USA) were collected: two (5 mL) containing sodium fluoride for plasma extraction, and two (10 mL) without content for serum extraction. The samples were immediately centrifuged at 800 RPM for 10 min and stored in aliquots of 1.5 mL at −20 °C. 

Plasma was used for glucose dosing by using an EON microplate spectrophotometer (Biotek Instruments Inc., Vermont, VT, USA) via the enzymatic method: glucose oxidase (Kovalent do Brasil Ltd., Rio de Janeiro, Brazil). Serum was used for insulin and insulin-like growth factor 1 (IGF-1) dosing. Analysis was carried out in the Immulite 2000^®^ (Siemens Healthineers, Brazil). The reagent Insulina 200T (IMM2) L2KIN2^®^ and control Biorad Liphocheck Control Trilevel Imuno^®^ were used for insulin, and the reagent IGF-1 200 Tests (IMM2)^®^ and control IGF1/IGFBP 3 (IMM)^®^ were used for IGF-1 (Prohosp Distribuidora de Medicamentos LTDA, Belo Horizonte, Minas Gerais, Brazil).

### 2.6. Infrared Thermography

Thermographic evaluations were carried out at 30, 60, and 90 d. Thermographic images were obtained with the support of the portable device, FLIR T420^®^ (FLIR Systems, Inc., Wilsonville, OR, USA). The distance from the thermograph to the photographed anatomical area was standardized at 1 m, and the device settings were adjusted to 20 °C reflectance temperature and 0.98 emissivity [11]. The archives were processed and interpreted by software FLIR Tools 5.6^®^ (FLIR Systems, Wilsonville, OR, USA) displayed on the iron palette. Measuring tools took the form of rectangles with different dimensions used in various anatomical areas: 40 mm × 30 mm for cheek, 150 mm × 120 mm for right rib, 10 mm × 10 mm for muzzle, 80 mm × 120 mm for left flank, 10 mm × 20 mm for front, 15 mm × 25 mm front limb, and 15 mm × 25 mm for hind limb. The evaluation was carried out using the maximum temperature of each area.

### 2.7. Statistical Analysis

The software SAS (SAS Institute Inc., Cary, NC, USA, version 9.4) was used. To evaluate the effects of efficiency in the groups, the MIXED procedure was used according to the model:

Y_ijk = β_0 + β_1 A_ij + β_2 B_ij + G_i + M_k + [GM]_ik + δ_ij + ε_ijk

where:

Y_ijk: dependent variable;
β_0: intercept;
β_1 A_ij: regression coefficient for the covariate initial BW;
β_2 B_ij: regression coefficient for the covariate total serum protein;
G_i: fixed effect of efficiency group;
M_k: fixed effect of repeated measure (day or week);
[GM]_ik: fixed effect of interaction between group and repeated measure;
δ_ij: random error between animals within treatment;
ε_ijk: random error between measurements among animals.

The mean, standard deviation, normality, and homoscedasticity were analyzed using the PROC-UNIVARIATE procedure. Abnormal variables were transformed according to their characteristics. Initial BW and total serum protein were tested as covariates and included in the model only if significant (*p* < 0.05). Initial body weight, final body weight, and ADG were analyzed using the PROC GLM. The remaining data were analyzed using repeated measures over time (PROC MIXED). HE-RFI or LE-RFI and repeated measures over time (day or week) were included in the model as fixed effects.

The PROC CORR procedure was used for the evaluation of the correlation between the variables. The correlations were classified as low (r < 0.30), moderate (0.30 > r > 0.70), or strong (r > 0.70). The *p*-value was considered significant when *p* < 0.05.

## 3. Results and Discussion

### 3.1. Residual Feed Intake, Feed and Water Intake, and Performance

A phenotypic divergence of DMI for RFI was observed between −0.358 and 0.337 kg/d for HE and LE, respectively (*p* < 0.0001) (Table 2). This variation represented a reduction of approximately 63.25 kg of DM for each animal during the trial period. The minimum and maximum values of RFI were −0.868 (HE) and 0.881 kg/d (LE), respectively, representing a difference of 1.75 kg/d of DM between the most or least efficient animals. The total DMI was 19.3% less in the HE animals (*p* < 0.0001). The difference was greater than the 15.9%, 13.0%, and 8.9% differences reported in other studies [9,11,17]. The first study was conducted with a Holstein × Limousin crossbreed (247 d of age) while the second and third evaluated Holstein × Gyr, and Gyr from birth to weaning. The major variation found in animals from 247 d and in the present study may be due to the life phase in which the evaluations were carried out. After weaning, the animals presented more advanced age and greater BW and were no longer subjected to a liquid diet, which may have allowed a major expression of variation in individual DMI. No difference was found in ADG between HE-RFI and LE-RFI in the present study. These results are in accordance with those reported by Basarab et al. [18] and McDonnel et al. [19]. 

To our knowledge, the present study is the first study to assess water consumption in phenotypically divergent dairy heifers for RFI. Water intake ranged between HE-RFI (7.2 L) and LE-RFI (7.0 L; *p* = 0.024) (Table 2). High-efficiency animals consumed proportionally more water in relation to their BW. Water intake is not a frequently measured parameter in studies and routines on farms, probably due to the difficulty of measuring. However, water is essential for the physiological process and is related to DMI, development, and ADG [20,21]. Increased water intake beyond that needed for organic functions does not reflect a higher DMI; nevertheless, when water intake is lower than the minimum required, it is followed by a reduction in DMI [22]. The ratio between water intake and DMI was 3.5 vs. 2.7 for HE-RFI and LE-RFI, respectively (*p* < 0.0001). The increase of water requirements does not keep up with body growth rate and body weight gain [23], as observed in our study, leading us to assume that greater water intake by HE could be related to some energetic metabolic pathway [21]. We believe that future studies on the biological bases involved in the phenotypic differences related to RFI should consider the assessment of water consumption, in order to elucidate the reasons for the higher water intake in HE animals.

### 3.2. Ingestive Behavior

Daily activity by animals is believed to contribute approximately 5% to the variation in RFI [7]. The values of total time at the bin and CTB were not different, as indicated in an earlier work [18]. However, NVB had less HE-RFI than LE-RFI (59 vs. 71; *p* < 0.001) (Table 3). Although moderate, except for the NVB, the other variables related to ingestive behavior showed no significance when correlated with RFI [23] (Table 4). 

HE-RFI animals can reach a difference of up to 22% in NVB [9,23]. In the present study, the difference was 16.9%. These findings show that the HE animals had less consumption time and consequently less intake in fewer visits and, as a result, fed more slowly and moved less often to go to the trough. Similar behavior was observed in Holstein heifers (5 to 9 months of age) [24]. Although our results are not in full agreement with previous studies, it is plausible to suppose, as suggested by these authors, that the HE groups possibly employed less energy in food events, so the energy saved could be used in other metabolic and physiological functions. 

Regarding water intake behavior, TTW (16 vs. 11 min) and NVW (5.9 vs. 5.5) differed between groups (*p* < 0.0001), being higher for HE-RFI animals (Table 3). In the present study, the HE animals consumed less food that was associated with higher TTW and water consumption, suggesting that the water intake is related to physiological functions in other tissues that contributed to the phenotypic divergence in RFI.

### 3.3. Ruminal Fermentation

We hypothesized that some changes could be observed between the groups, since part of the variation in feed efficiency is related to digestion and energy metabolism [25], but this was not confirmed by the results, because no differences were observed in any ruminal fermentation variables (Table 3). Guan et al. [26] observed a higher concentration of butyrate in HE-RFI animals and a higher concentration of total VFA and acetate in LE-RFI animals (*p* = 0.059 and *p* = 0.074). Those authors suggested that there was increased microbial activity in HE-RFI animals. However, as in the present study, no variation in the ruminal fermentation profile was found by other authors [2,27]. Nevertheless, according to Trevizan et al. [2], other factors may have greater relevance to the efficiency of these animals than the ruminal fermentation profile. In this sense, Elolimy et al. [5] found different profiles of microorganisms and different metabolic potentials associated with metabolites of energetic pathways between low and high-efficiency Holstein heifers at birth and during preweaning.

For rumen functioning, factors such as food, water, and microorganisms are necessary, and the variation in their quantity and quality may interfere with ruminal parameters as well as with their development [22]. However, the variation in the concentration of VFA is most evident in the preweaning phase, when intense changes in rumen development occur. Heifers in this study received a solid diet of high granulometry and hay while still in the preweaning phase, which may have had a positive effect on most of the rumen development in this phase of life, causing the animals to reach postweaning with similar ruminal developed.

A study of dairy cows found that more efficient animals presented lower amylase, cellulase, and protease activity, which was attributed to the DMI decreased [28]. The authors believe that the lower DMI allowed animals with HE to have a longer time for digestion, requiring less food to support their maintenance. Together with the microbial profile, these results show that, because they are closely related to DMI, the most varied conditions of the rumen environment can be associated with the RFI phenotype.

### 3.4. Blood Metabolites and Hormones

No differences were observed in glucose, insulin, and IGF-1 concentrations, and the glucose:insulin ratio between HE and LE groups for RFI (Table 3), was corroborated in research that found no difference in glucose concentration between HE-RFI and LE-RFI [29]. However, a moderate correlation was observed between glucose and DMI (0.33; *p* = 0.0017) and between glucose and ADG (0.35; *p* = 0.0009) (Table 4). These findings were similar to those of other works that reported the significant effects of glucose concentrations on DMI and ADG [9,30]. As suggested in those works, this effect was likely the result of the physiological increase in DMI as the age of the animal advances. Diet type and composition may affect the way IGF-1 and RFI relate to each other and may generate positive correlations between IGF-1 and RFI in high roughage diets as well as negative correlations when using highly concentrated diets [31]. In our study, as with glucose, IGF-1 had a moderate correlation with DMI (0.30; *p* = 0.0066) and ADG (0.31; *p* = 0.0035). However, a correlation between IGF-1 and RFI was not observed, contrary to in the results of another work that reported a moderate correlation between both [32]. DMI elevates the insulin concentration in response to nutrient absorption by the gastrointestinal tract. In the present study, a divergence in insulin concentration was expected to be found that would demonstrate the possible superiority of metabolic efficiency of HE-RFI. Nevertheless, this hypothesis was not confirmed since unlike glucose and IGF-1, the insulin and glucose:insulin ratio did not correlate with DMI and ADG. We do not recommend their use when studying phenotypic divergence in RFI for heifers.

### 3.5. Infrared Thermography

No differences were found in infrared thermography between HE-RFI and LE-RFI. However, although there was no difference between the groups considering the entire evaluation period, a difference was detected in only the infrared thermography at 30 d. The maximum left flank temperature at 30 d was 36.6 °C for HE-RFI and 34.8 °C for LE-RFI (*p* = 0.0292) and the muzzle was 31.9 °C for HE-RFI and 29.1 °C for LE-RFI (*p* = 0.0218) (Table 3). In previous works on dairy cattle, authors reported greater temperatures on the udder in LE-RFI adult animals [33]. The researchers suggested that HE-RFI animals would have greater energy efficiency and thus lower surface temperatures. In contrast, other works reported higher surface temperatures in HE-RFI animals in beef cattle [10,34]. Those authors suggest that greater feed-efficient animals had higher surface temperatures which were related to their metabolic efficiency [35], which led to greater heat dissipation by radiation. Although differences were observed at 30 d for the HE and LE groups, the present study does not support the idea that infrared thermography would be a potential tool for finding RFI phenotypic divergence in heifers due to the weaker correlation between the thermographic variables and the classifications of these indexes.

## 4. Conclusions

Phenotypic divergence in RFI was observed in the dairy heifers studied. Feed intake was the best predictor of said divergence. Water intake and the number of visits to the feed bin presented moderate correlations with RFI. Ruminal and blood parameters, ingestive behavior, and infrared thermography evaluated in this work were not good predictors of phenotypic divergence for RFI in dairy heifers. Future studies should be conducted to explain the relationship of these variables to the biological bases that lead to the differences in feed efficiency in animals.

## Figures and Tables

**Table 1:** Nutritional composition (dry matter basis, % unless otherwise noted) of concentrate, corn silage and total mixed ration offered to heifers during trial period.

Nutritional Composition	Concentrate ^1^	Corn Silage	TMR ^2^
DM ^3^	88.45	35.10	48.40
TDN ^4^	85.10	75.80	77.20
CP ^5^	33.31	8.10	14.41
EE ^6^	2.96	4.00	3.76
ASH ^7^	11.87	6.10	7.54
NDF ^8^	11.69	42.90	35.12
ME ^9^ (kcal/kg)	3591.00	2240.00	2577.00
GE ^10^ (kcal/kg)	4205.84	4510.50	4433.57

^1^ Concentrate composition: 64% soybean meal, 30% ground corn and 6% mineral core; ^2^ TMR = total mixed ration; ^3^ DM = dry matter; ^4^ TDN = total digestible nutrients; ^5^ CP = crude protein; ^6^ EE = ethereal extract; ^7^ ASH = ashes; ^8^ NDF = neutral detergent fiber; ^9^ ME = metabolizable energy; ^10^ GE = gross energy.

**Table 2:** Means of the indexes, intakes, and performances evaluated for HE and LE in residual feed intake (RFI); standard error of the mean (SEM) and group interaction (high efficiency: HE or low efficiency: LE) in dairy heifers phenotypically classified for RFI.

Item	RFI ^1^		SEM ^4^	*p*-Value
	HE ^2^	LE ^3^		
RFI (kg/d)	−0.358	0.337	0.06	<0.0001
Intake				
Water (L/d)	7.2	7.0	0.50	0.024
DMI ^5^ (kg/d)	2.5	3.1	0.17	<0.0001
Water: DMI ratio	3.5	2.7	0.30	<0.0001
Performance				
ADG ^6^ (kg/d)	0.305	0.237	0.35	0.3662
Initial weight body (kg)	106.3	107.6	1.88	0.8214
Final weight body (kg)	133.7	128.9	4.22	0.4682

^1^ RFI = residual feed intake; ^2^ HE = high efficiency (RFI smaller than 0.5 SD below average); ^3^ LE = low efficiency (RFI greater than 0.5 SD above average); ^4^ SEM = standard error of the mean; ^5^ DMI = dry matter intake; ^6^ ADG = average daily gain.

**Table 3:** Ingestive behavior, ruminal fermentation, blood parameters and infrared thermography in HE and LE dairy heifers phenotypically classified for RFI.

Item	RFI ^1^		SEM ^4^	*p*-Value
	HE ^2^	LE ^3^		
Ingestive behavior				
TTB ^5^ (min)	152	152	8.27	0.36
CTB ^6^ (min)	126	126	7.90	0.14
NVB ^7^ (occurrence)	59	71	0.80	<0.0001
TTW ^8^ (min)	16	11	0.06	<0.0001
NVW ^9^ (occurrence)	5.9	5.50	0.34	<0.0001
Ruminal parameters				
pH	6.80	6.90	0.16	0.74
N-NH_3_^10^ (mg/dL)	13.70	14.50	1.17	0.25
Acetate (µmol/mL)	41.60	43.10	3.34	0.45
Butyrate (µmol/mL)	8.70	8.80	0.86	0.72
Propionate (µmol/mL)	7.90	7.80	0.66	0.91
Acetate:propionate	5.30	5.20	0.41	0.60
Total VFA (µmol/mL)	58.80	60.40	4.45	0.53
Blood parameters				
Glucose (mg/dL)	78.80	80.20	2.98	0.42
Insulin (uIU/mL)	11.10	11.30	2.37	0.89
IGF-1 ^11^ (ng/mL)	82.90	89.90	18.31	0.47
Ratio glucose:insulin	9.20	9.50	1.83	0.72
Infrared termography (°C)				
Cheek	35.30	34.70	0.45	0.37
Right rib	35.70	35.20	0.68	0.71
Left flank	36.60	34.80	0.82	0.08
Front	33.20	31.40	1.30	0.24
Muzzle	31.90	29.20	1.27	0.26
Front limb	35.30	33.00	1.29	0.34
Hind limb	30.80	29.70	1.28	0.50

^1^ RFI = residual feed intake; ^2^ HE = high efficiency; ^3^ LE = low efficiency; ^4^ SEM = standard error of mean; ^5^ TTB = total time at the bin; ^6^ CTB = consumption time at the bin; ^7^ NVB = number of visits to the feed bin with consumption; ^8^ TTW = total time at the water bin; ^9^ NVW = number of visits to the water bin; ^10^ N-NH_3_ = ammonia nitrogen content; ^11^ IGF-1 = insuline-like growth factor 1.

**Table 4:** Phenotypic correlation of dry matter intake (DMI), average daily gain (ADG), and RFI with the performance indexes and evaluated variables in dairy heifers phenotypically classified for RFI.

Item	DMI ^1^	ADG ^2^	RFI ^3^
Intake/Performance			
RFI	-	-	-
DMI	-	0.60 ***	0.78 ***
Water intake	-	0.50 **	-
ADG	0.60 ***	-	-
Initial body weight	0.37 *	0.57 **	-
Final body weight	0.56 **	0.91 ***	-
Ingestive behaviour			
TTB ^4^	-	0.06 **	-
CTB ^5^	-	0.06 **	-
NVB ^6^	0.43 *	-	0.38 *
TTW ^7^	0.18 ***	0.18 ***	−0.13 ***
NVW ^8^	0.18 ***	0.18 ***	0.04 *
Blood variables			
Glucose	0.33 **	0.35 **	-
IGF-1 ^9^	0.30 **	0.31 **	-

^1^ DMI = Dry matter intake; ^2^ ADG = Average daily gain; ^3^ RFI = Residual feed intake; ^4^ TTB = Total time at the bin; ^5^ CTB = Consumption time at the bin; ^6^ NVB = Number of visits to the feed bin with consumption; ^7^ TTW = Total time at the water bin; ^8^ NVW = Number of visits to the water bin; ^9^ IGF-1 = Insuline-like growth factor 1; * *p* < 0.05; ** *p* < 0.01; *** *p* < 0.0001 and (-) no significance.

## Data Availability

The data that support the findings and which are presented in this study are available on reasonable request from the corresponding author, Mariana Magalhães Campos, mariana.campos@embrapa.br. The data are not publicly available as not all data of the study has been published yet.
